# Small Bowel Obstruction Secondary to a Metamucil Bezoar: Case Report and Review of the Literature

**DOI:** 10.1155/2017/2702896

**Published:** 2017-09-20

**Authors:** Sara Abou Azar, Mohammad Rachad Wehbe, Sarah Jamali, Ali Hallal

**Affiliations:** Department of Surgery, American University of Beirut Medical Center, Cairo Street, Riad El Solh, P.O. Box 11-0236, Beirut 1107 2020, Lebanon

## Abstract

Bezoar-induced small bowel obstruction is a rare entity. It should be highly suspected in patients with gastric hypomotility disorders, psychiatric conditions, prior abdominal or bariatric surgery, or improper intake of medication. Their diagnosis is quite challenging and surgical exploration remains the best treatment of choice to ensure the viability of the small bowel tissue and relieve the obstruction. This is a case of a 48-year-old female with no previous abdominal surgery who presented with acute abdominal pain. The patient's history was remarkable for the daily ingestion of 1.5 teaspoons of Metamucil with minimal amount of water. Computed tomography scan demonstrated dilated small bowel loops and a transition zone at the level of the mid jejunum. On laparoscopy, the patient was found to have a hard mass in the mid jejunum amenable to gentle fragmentation and breakdown. Metamucil bezoars are due to the solidification of psyllium-based substances in the gastrointestinal tract. The usual management of small bowel obstruction induced by a bezoar is exploratory laparotomy with enterotomy and primary anastomosis. Laparoscopic intervention has gained popularity among surgeons with good outcome and lower morbidity. In this unusual case, the small bowel obstruction induced by the Metamucil bezoar was safely treated with laparoscopic fragmentation alone.

## 1. Introduction

Bezoars are insoluble conglomerations that persist in the gastrointestinal tract. Although rare, they are usually reported in patients who have undergone previous partial gastrectomies, pyloroplasties, and vagotomies due to delayed gastric emptying and impaired motility [1]. Phytobezoars, made up of undigested plant products, are identified as the etiologic cause of approximately 4% of cases of intestinal obstruction [1]. Intestinal obstruction secondary to ingestion of pharmaceutical agents or nutritional supplements is extremely rare. We report a rare case of an intestinal obstruction secondary to ingestion of Metamucil (a psyllium-based bulk-forming laxative).

## 2. Case Report

A 48-year-old female presented to the Emergency Department at American University of Beirut Medical Center in August 2016 with severe and persistent crampy abdominal pain of 18 hours duration. The abdominal pain was focused around the supraumbilical region. It was associated with obstipation but no abdominal distention. She denied any fever, chills, nausea, vomiting, diarrhea, or any respiratory and cardiac symptoms. Her past medical history was only significant for a pituitary microadenoma and migraine headaches. Her past surgical history was significant for a Caesarean section and an abdominoplasty. She regularly took Imigran (Sumatriptan) 50 mg and Metamucil fiber supplements (1.5 spoons). She mentioned the ingestion of 1.5 spoons of Metamucil dissolved in a minimal amount of water.

On admission, her vitals were stable: temperature 36.4°C, heart rate 63 beats per minute, blood pressure 130/80 mmHg, and oxygen saturation 96% on room air. Physical examination showed mild diffuse abdominal tenderness and hypoactive bowel sounds. There was no guarding and rebound tenderness. Murphy and McBurney signs were negative. An abdominoplasty scar was noted in the lower quadrant of the abdomen. Laboratory data were grossly normal with no evidence of electrolyte abnormalities or increased white blood cell count.

Computed tomography (CT) of the abdomen and pelvis with IV and oral contrast was performed in the emergency department. Oral contrast was seen reaching the proximal jejunal loops. There was evidence of dilated duodenal and jejunal loops reaching a maximum diameter of 3.6 cm with small bowel feces sign proximal to a transition zone at the level of the mid jejunum. Distal jejunal and ileal loops were collapsed. These findings were suggestive of high-grade small bowel obstruction with a clear transition zone at the level of the mid jejunum.

**Figure 1:**
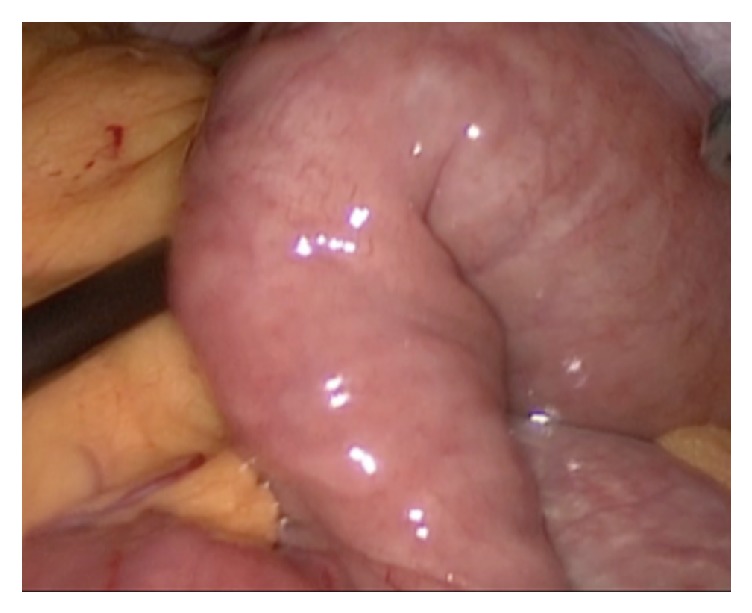
Transition zone in mid jejunum.

**Figure 2:**
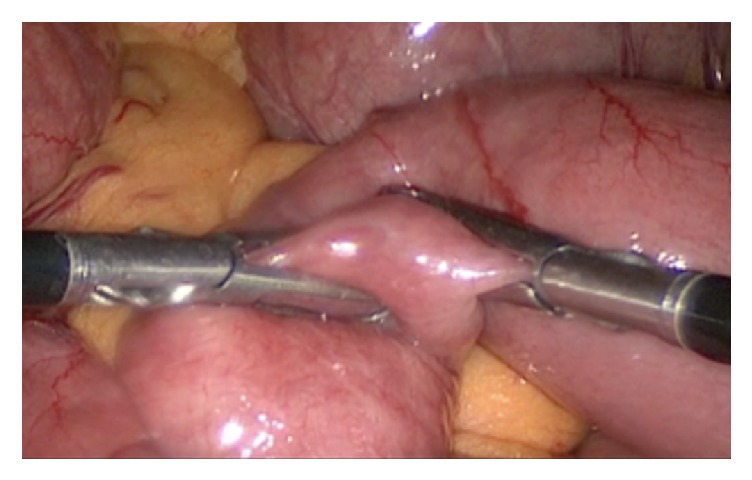
Gentle laparoscopic manipulation and fragmentation.

The patient consented for laparoscopic exploration. Laparoscopic exploration revealed a clear transition point in the mid jejunum (see Figure 1). No adhesions, masses, or abnormalities were noted in the bowel and mesentery that could explain the pathology. Upon palpation of the transition zone, an area of rigid bowel contents was felt within the bowel loops suggestive of a bezoar (see Figure 2). The bezoar was soft enough to be amenable to manual breakdown by crushing it using the laparoscopic instruments. The option of performing a minilaparotomy to exteriorize the bowel and an enterotomy to evacuate the bezoar was entertained; however, the bolus of undigested material was not large and successfully broken down. In addition to that, the degree of obstruction was not severe so it was decided not to perform an enterotomy.

The patient's postoperative course was smooth and facilitated by the use of a mild dose of polyethylene glycol. The patient's crampy abdominal pain resolved and she showed evidence of return of bowel function (flatus and stool) on postop day 1. Oral intake was resumed postop day 1 and advanced accordingly. The patient was discharged home after 2 days uneventfully and was doing well at the time of her last follow-up in clinic.

## 3. Discussion

Bezoars can be classified into four different entities according to their composition: trichobezoars, phytobezoars, lactobezoars, and pharmacobezoars [2–6]. Bulk-forming laxatives such as perdiem, psyllium, and guar gum have been implicated in the formation of the latter due to their ability to attract water molecules and stagnate in the lumen of GI tract [2]. Metamucil is a bulk-forming laxative composed of psyllium. It is a fiber used for restoring and maintaining bowel regularity. Usual recommended dosage is one rounded teaspoonful in 8 ounces of liquid. In our case, the patient reported that she had been taking a large amount of this supplement daily dissolved in minimal amount of water. Metamucil bezoars as the cause of SBO are extremely rare. An extensive literature review identified a reported case of SBO caused by a Metamucil bezoar, published in 1992 by Frohna [7]. The obstruction was located at the gastric outlet and proximal small bowel and was relieved by nasogastric decompression.

Depending on size and location, some bezoars may remain undetected, while others show gastrointestinal signs and symptoms (bloating, abdominal pain, GI bleeding, nausea, vomiting, or weight loss) [8]. They can also lead to GI obstruction and ileus [6]. Around 4% of small bowel obstructions (SBO) are due to bezoars in the GI tract [1]. Prompt diagnosis of bezoars is crucial as they can cause life-threatening complications such as gastrointestinal ulcerations and pressure necrosis [3, 8].

This can be achieved through thorough history taking, physical examination, endoscopy, and computed tomographic (CT) scanning. The latter allows localization and assessment of the presence of multiple bezoars and can detect the cause of the obstruction in around 73–95% of patients [6]. There have been several modalities in the literature for the diagnosis and treatment of GI bezoars. In 2012, a systemic review done by Ladas et al. demonstrated that Coca-Cola nasogastric lavage alone led to a 50% complete resolution of gastric phytobezoars and the remaining cases required either further endoscopic intervention (41.3%) or surgery (8.7%) to achieve complete dissolution of the bezoar [9]. However, in a study done by Lee et al. on 17 cases of gastric phytobezoars, only 4 patients had complete resolution with the use of Cola lavage (23.5%). The authors recommended this technique only be used as pretreatment to make fragmentation easier during endoscopy or surgery [10]. Alternative treatment products, including papain (enzyme extract), cellulase, and prokinetic agents, have not been shown to be very effective [3].

Endoscopy has been widely used for fragmentation and/or extraction of gastric bezoars using forceps, snares, argon plasma coagulation, or electrohydraulic devices [3]. Some bezoars may need multiple endoscopies to be completely fractured due to their “hard consistency.” Trichobezoars may be especially difficult to remove endoscopically and may necessitate surgery. Although potentially cost-effective, endoscopic treatment of intestinal bezoars causing obstruction is challenging and rarely reported. This may be due to inaccessibility, technical difficulties, and risk of perforation, especially when the bezoar is adherent to the bowel wall [11].

Surgical intervention remains the gold standard for the treatment of bezoar-induced SBO and ileus [12]. The optimal surgical management of small intestinal bezoars is exploratory laparotomy with enterotomy and evacuation. Inspection of the abdomen is performed intraoperatively to detect the presence of multiple gastric or bowel bezoar [12]. Treatment options vary according to perioperative findings. For example, bowel ischemia and necrosis may direct the surgeon towards segmental resection and anastomosis. Laparoscopy can also be used and is associated with reduced morbidity. In our case, tissue necrosis was absent and the bezoar was amenable to fragmentation. This allowed us to spare the patient an enterotomy, while we considered the risk of the bezoar reform further down in the gastrointestinal tract.

## 4. Conclusion

Pharmacobezoars are rare causes of small bowel obstruction, especially those composed of Metamucil and psyllium-based substances. The diagnosis is challenging and patient symptoms vary with the severity of the obstruction. Gastric hypomotility disorders increase the risk of bezoars and timely intervention is critical to prevent tissue ulceration and necrosis. Laparoscopic fragmentation of the bezoar if feasible is an acceptable option sparing the patient the risk of an enterotomy.
